# Unraveling Type 1 Diabetes: Integrating Microbiome, Metabolomics, and Immunomodulation for Next-Generation Therapies

**DOI:** 10.3390/ijms262110788

**Published:** 2025-11-06

**Authors:** Pleun de Groen, Lente C. H. M. Blok, Coco M. Fuhri Snethlage, Nordin M. J. Hanssen, Elena Rampanelli, Max Nieuwdorp

**Affiliations:** 1 Department of Internal and Vascular Medicine, Amsterdam UMC, University of Amsterdam, 1105 AZ Amsterdam, The Netherlands; p.degroen@amsterdamumc.nl (P.d.G.); l.c.blok@amsterdamumc.nl (L.C.H.M.B.); c.m.fuhrisnethlage@amsterdamumc.nl (C.M.F.S.); n.m.j.hanssen@amsterdamumc.nl (N.M.J.H.); e.rampanelli@amsterdamumc.nl (E.R.); 2 Amsterdam Diabeter Center, 1066 EC Amsterdam, The Netherlands

**Keywords:** type 1 diabetes, microbiome, metabolomics, immunomodulation

## Abstract

Type 1 diabetes (T1D) is a chronic autoimmune disease characterized by T-cell-mediated destruction of pancreatic beta cells, resulting in insulin deficiency. Both genetic predisposition and environmental factors contribute to T1D development, with growing evidence implicating the gut microbiome as a critical environmental modulator in disease pathogenesis. Gut microbial composition and derived metabolites influence immune homeostasis and autoimmunity. This review summarizes recent advances elucidating immune dysregulations in T1D and novel therapeutic strategies to preserve beta cell function. We discuss approaches such as immune cell engineering, including CAR-Treg therapy, and targeted modulation of immune signaling pathways like JAK-STAT. Furthermore, we explore the role of the gut microbiota and its metabolites in modulating host immunity and describe emerging microbiome-targeting interventions, including fecal microbiota transplantation and metabolite supplementation. These interventions show promise in modulating disease progression in preclinical and early clinical studies. An integrated understanding of immune and microbiome-related mechanisms is critical for developing next-generation therapies. Further research and clinical trials are needed to optimize these approaches and translate them into durable, personalized treatments for individuals with T1D.

## 1. Introduction

Type 1 diabetes is a devastating autoimmune disease—characterized by the destruction of insulin-producing beta cells in pancreatic islets, resulting in hyperglycemia—that affects 8.75 million people worldwide [1,2,3]. The disease can manifest at any age, and even though 50% of new-onset patients are adults, type 1 diabetes is the most prevalent autoimmune disease in children and adolescents [2]. The incidence of type 1 diabetes is increasing, and by 2040, the prevalence is projected to reach 13.5–17.4 million [2]. This is alarming, as people living with type 1 diabetes depend on a complex regimen of lifelong exogenous insulin therapy, and disease management is constant and difficult, with a high psychological and sociological impact on quality of life [4]. Alongside the high psychological burden, the metabolic repercussions accompanying deregulated insulin secretion extend beyond hyperglycemia and accelerate cardiovascular disease, resulting in a five-times elevated risk of developing cardiovascular events and increased cardiovascular and all-cause mortality, with a reduced lifespan of 13 years [5,6,7].

There is a genetic susceptibility to type 1 diabetes, mostly mapped to human leukocyte antigen (HLA) types, which facilitate antigen representation to T cells [8]. HLA-DR and HLA-DQ have the strongest association with the development of type 1 diabetes, but the aforementioned increase in the incidence of type 1 diabetes is not explained by genetics alone, and indeed, people with less-susceptible HLA types increasingly develop the disease [9,10].

Currently, type 1 diabetes remains incurable; nonetheless, the landscape for insulin administration has vastly changed in the last few decades, and treatment of type 1 diabetes has greatly improved with the emergence of new technological advancements such as continuous glucose monitoring [CGM], insulin pumps, and automated insulin delivery [AID] systems or hybrid closed-loop systems [11,12,13]. Moreover, after a decades-long lull following the discovery of insulin over a century ago, multiple therapeutic milestones have been reached in medical efforts to preserve beta cell function and delay the onset of type 1 diabetes [14]. This is in part due to a better understanding of the immunobiology of autoimmunity in type 1 diabetes and the development of novel immunotherapies, without chronic immunosuppression. However, although new approaches may delay type 1 diabetes onset and beta cell decay, they do not completely halt insulin deficiency in type 1 diabetes indefinitely.

This review aims to provide an overview of recent exciting developments in next-generation therapies in the field of type 1 diabetes, specifically focusing on (microbiome-based) immune-modulatory therapies, as well as microbiome- and metabolite-driven strategies.

## 2. Pathophysiology of Type 1 Diabetes: Immune Modulators and Therapeutic Targets

Although type 1 diabetes in not thought to be an autoantibody-mediated disease, the onset of hyperglycemia is preceded by seroconversion to type 1 diabetes-specific autoantibodies against glutamic acid decarboxylase (GADA), insulin (IAA), islet antigen 2 (IA-2A), or the ZnT8 transporter (ZnT8A), followed by immune cell infiltration of the pancreatic islets, resulting in insulitis [15,16,17]. Although autoantibodies are thought not to be pathogenic in autoimmune T1D, B cells play an important role mainly as antigen-presenting cells [18,19]. Indeed, studies have shown that loss of B cell antigen presentation can prevent T1D onset in non-obese diabetic (NOD) mice [18,19,20].

Although CD8 T cells constitute the majority of islet-infiltrating lymphocytes in human T1D [21] and are directly responsible for beta cell killing [22,23], their activation depends on other immune cells, namely, antigen-presenting cells and CD4 T helper (Th) cells. Indeed, through interactions with CD4 helper T cells, dendritic cells fully acquire the cross-presenting antigen capacity, allowing them to activate CD8 cytotoxic T cells [24,25]. Among the antigen-presenting cells, macrophages are abundantly found in inflamed islets, and macrophage depletion studies on mice have shed light on their crucial role in autoimmune diabetes by promoting CD8 T cells infiltration of islets and beta cell killing [25,26].

**Figure 1:**
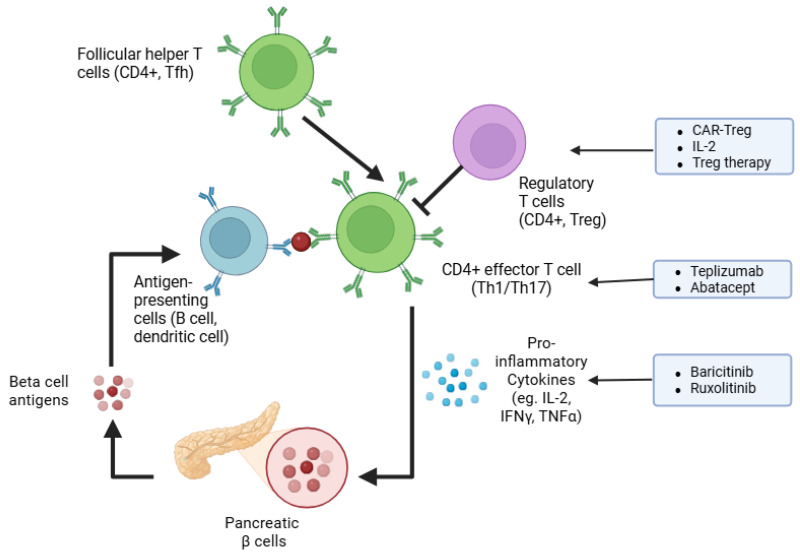
Emerging immunotherapy strategies to halt autoimmunity in T1D. The schematic illustrates key steps in immune-mediated destruction of pancreatic β cells, including the roles of antigen-presenting cells (APCs), follicular helper T cells (Tfh), effector CD4+ T cells (Th1/Th17), and regulatory CD4+ T cells (Treg). Broad nonspecific approaches, such as antibody-based therapies (e.g., teplizumab, abatacept), cytokine signaling inhibitors (e.g., baricitinib, ruxolitinib), and cytokine modulation are highlighted. Specific tolerance to islet antigens is addressed by regulatory T cell-based therapies (e.g., CAR-Treg, low-dose IL-2).

Islet-reactive CD4 T cells, such as follicular helper Tfh cells, Th17, and Th1 T-helper cells, have all been implicated in the pathogenesis of this disease [27,28,29]. The importance of helper CD4 T cells in T1D pathophysiology is proven by depletion studies. For instance, in NOD mice, depletion of CD4 T cells caused by monoclonal antibodies cured diabetes when administered shortly after hyperglycemia onset [30]. In line, one course of teplizumab—humanized anti-CD3 monoclonal antibodies causing reversible lymphodepletion—delayed the onset of T1D in individuals at risk of T1D development and preserved beta cell function in new-onset T1D patients, although it did not improve time in the euglycemic range or glycated hemoglobulin levels [14,31]. Figure 1 summarizes the immunologic pathways implicated in T1D and highlights current and emerging molecular targets for immunotherapy.

Tepluzimab is the first FDA (USA Food and Drug Administration)-approved disease-modifying drug for delaying clinical type 1 diabetes [32] (Figure 1). Similarly, modifying T cell activation has also shown promising results. Trials employing treatment with abatacept, which blocks CD80/CD86-dependent T-cell co-stimulation (the second signal required for T-cell activation), have shown that abatacept slows the decline in beta cell function in new-onset T1D patients in the first 2 years of treatment and impact the CD4 T memory T cell compartment by reducing effector T cells and, specifically, follicular helper T cells [33,34,35]. However, unlike tepluzimab, abatacept treatment could not delay the progression to glucose intolerance in autoantibody-positive at-risk individuals [35] (Table 1).

The frequencies of activated memory CD4 T cells, reactive to PPI, GAD65, ZnT8, IGRP, or ChgA, were found to be elevated in the circulation of individuals with T1D [67]. Instead, studies investigating the population of immunosuppressive regulatory CD4 T cells (Tregs) in T1D have disclosed inconsistent results, with some reporting a reduction and no other change in frequency in the blood of patients with T1D [38,39]. Nonetheless, several investigations have demonstrated a defective function in the Tregs from T1D patients, with an impaired ability to suppress T cell proliferation [40,41,42].

Murine studies with depletion of Tregs have proven their importance in limiting autoimmune diabetes and insulitis [43,44]. Conversely, adoptive transfer of Tregs in NOD mice can reverse diabetes [45]. In the same vein, in newly diagnosed diabetic children, infusion of autologous Tregs preserves beta cell function and lowers the requirements of exogenous insulin [46]. With a different approach, Rosenzwajg and colleagues administered low doses of IL-2 to selectively activate and expand the endogenous Treg population in new-onset T1D children. Although they observed a certain degree of individual variability in IL-2-induced Treg responses, they found that children with high Treg expansion also displayed a better preservation of insulin secretion, underscoring the role of Tregs in limiting autoimmunity [47]. In a later trial, low-dose IL-2 was combined with polyclonal Treg adoptive therapy based on the assumption that IL-2 would expand the exogenously administered Treg cells; however, the investigators disclosed that in addition to an augmented Treg frequency, cytotoxic CD8 T cells were also substantially increased, calling for caution in the use of exogenous IL-2 in T1D [48].

Exciting advances have been made over the last decade in the field of immune cell engineering and the generation of CAR (chimeric antigen receptor) T cells, which provide the advantage of antigen-specificity. CAR-Treg therapy could potentially enable targeted immunotolerance against islet-derived autoantigens and, therefore, achieve long-lasting remission of T1D. In murine studies, adoptive transfer of CAR-Tregs specific for the insulin B chain 10-23 peptide prevented diabetes [49]. Interestingly, another study showed that Treg engineered to express a T cell receptor (TCR) specific for the glucose-6-phosphatase catalytic subunit-related protein (IGRP) peptide can suppress islet-specific effector T cells specific for the same antigen, and they have a bystander suppression of effector T cells, recognizing different islet epitopes [50]. This suggests that CAR-Treg therapy may induce broad immunosuppression against polyclonal islet-specific pathogenic T cells. Nonetheless, immune cell therapy faces major challenges, including the costs due to the complexity of manufacturing and administration, as well as safety issues due to the possible off-targeting effects of genetic engineering, which, overall, may limit broad accessibility to T1D worldwide [51]. In this regard, therapeutic strategies targeting immune signaling pathways pivotal for immune cell function represent a more accessible and feasible approach. For instance, the Janus kinase (JAK)–signal transducer and activator of transcription (STAT) pathway orchestrates lymphocyte growth, immune function, and cytokine signaling, and it also induces the overexpression of HLA class I molecules by beta cells, thereby facilitating islet-derived autoantigen presentation [52]. As it is hyperactivated in chronic inflammatory and autoimmune conditions, targeting the JAK-STAT pathway may mitigate the autoimmune attack directed to beta cells in T1D [53]. Recently, daily treatment of new-onset T1D patients for 48 weeks with baricitinib, a JAK1 and JAK2 inhibitor, resulted in preserved beta cell function, assessed by mixed-meal tests, reduced glycemic variability, and proportion of circulating memory effector CD8 T cells [54]. In addition, a case report disclosed that therapy with the JAK1/2 inhibitor ruxolitinib resolved autoimmune diabetes, caused by a STAT1 gain-of-function mutation, and led to the discontinuation of exogenous insulin administration after 12 months of ruxolitinib therapy [55]. Although JAK-STAT pathway inhibitors hold promising effects, we are still waiting for long-term safety data on adverse events and risks of infections or malignancies.

Altogether, to advance targeted strategies to halt T1D progression at different stages of beta cell destruction, further research and clinical trials are necessary to better understand the pathophysiology of type 1 diabetes, as it is a complex and heterogeneous disease [16].

Finally, although immune cells are actively involved in beta cell destruction, through cross-talk with immune cells and/or intrinsic dysfunction, beta cells can contribute to T1D development [16]. For instance, beta cells can generate neoantigens through diverse peptide modifications, such as oxidation of insulin chains or post-translational generation of hybrid insulin peptides (insulin fragments fused with other peptides) [56,57,58,59,60,61]. In addition, inflammatory conditions, oxidative stress, endoplasmic reticulum stress, and senescence have been shown to contribute to T1D pathophysiology and killing by T cells, which can be activated by dying or senescent beta cells [68,69,70,71].

Importantly, the underlying drivers of T1D pathophysiology comprise both genetic and environmental factors. Indeed, the growing worldwide incidence of diabetes and the notion that the monozygotic concordance rate is approximately 50% indicate that environmental triggers induce T1D onset [72,73,74]. Multiple such triggers have been suggested, including dietary components such as gluten; decreased vitamin D levels; and viral pathogens such as infections with enterovirus, rotavirus, and norovirus [17,62,63,64,65]. Notably, both the occurrence and the absence of infections have been indicated as risk factors for autoimmune diabetes. According to the “hygiene hypothesis”, our immune systems have evolved to protect us from the constant threat of pathogenic infections, and encountering diverse microorganisms in early life is important to educate our immune systems and promote immune tolerance to self-antigens [37]. Throughout life, we are constantly exposed to commensal bacteria, and accumulating evidence indicates that commensal microorganisms and, specifically, the gut microbiota can modulate host immune responses and are implicated in the pathogenesis of type 1 diabetes [36]. Changes in the composition of the gut microbiome have been observed in individuals with type 1 diabetes even before the onset of autoantibody seroconversion in at-risk children [17]. This suggests that disruptions in the gut microbiota may precede and potentially contribute to disease development. The gut microbiota also contributes to upholding the intestinal barrier, and disruption can result in systemic release of bacterial metabolites and endotoxins. Increased intestinal permeability and reduction in the gut barrier, commonly referred to as “leaky gut,” may facilitate immune activation and systemic inflammation in type 1 diabetes [66].

In the next sections of this review, we will elucidate how gut microbiome composition and function are linked to T1D and can modulate disease progression, and we summarize the results of microbiome-targeting therapeutic approaches in human and murine T1D.

## 3. Alterations in the Gut Microbiome Associated with T1D

The gut microbiome (comprising bacterial strains, viruses, phages, and fungi) is defined as a complex ecosystem comprising approximately 40 trillion microbial cells, a number comparable to that of human cells [75,76]. While previously regarded as a harmless or symbiotic collaborator, recent research has unveiled the gut microbiome’s active involvement in regulating host metabolism and immunity, particularly through metabolite-mediated mechanisms [75,77]. Early-life microbial colonization occurs alongside the maturation of the host immune system, and disruptions in early-life microbiota induced by antibiotic use, caesarean delivery, or variations in infant feeding have been implicated in the pathogenesis of type 1 diabetes (T1D) [17,78,79,80]. The perturbations in gut microbiota composition (which are only the bacterial strains) and function (often referred to as dysbiosis) are mainly characterized by a decline in microbial diversity, the overgrowth of pathobionts, and the disruption of community balance. Dysbiosis has been progressively acknowledged as a contributing factor to the autoimmune processes underpinning T1D. Although numerous studies have investigated the microbial signatures associated with type 1 diabetes, there is currently no consensus on specific microbial species that consistently drive or predict disease onset [81,82].

Nonetheless, a common finding is a significant reduction in both the alpha and beta diversity of the gut microbiota in individuals with type 1 diabetes compared to healthy controls [83,84].

Due to the practical and ethical limitations imposed on human studies, murine models, most notably the non-obese diabetic (NOD) mouse, have played an instrumental role in elucidating the mechanistic correlations between the gut microbiome and the onset of type 1 diabetes (T1D). NOD mice spontaneously develop autoimmune diabetes and share key features with human T1D, including genetic susceptibility, autoantibody production, and autoreactive T cell responses. Murine models also permit extensive manipulation of the gut microbiome and of the murine genetic background to unravel the microbiome-immune cross-talk that may play a role in inflammation and immune tolerance [85].

By comparing the microbiome of NOD mice with the microbiome of diabetes-resistant non-obese (NOR) mice, which do not develop insulitis, several studies have identified distinct microbial signatures. For instance, the ileal communities of NOD mice exhibit diminished levels of protective Lactobacillus species and segmented filamentous bacteria, accompanied by elevated pro-inflammatory *Desulfovibrio* populations [86,87]. In the colon, NOD mice exhibit a lower prevalence of *Alphaproteobacteria*, *Bacteroides acidifaciens*, and *Ruminococcus gnavus*, accompanied by a higher abundance of *Prevotella*. It is noteworthy that the transplantation of fecal microbiota from NOD mice into NOR mice has been shown to induce insulitis, thereby underscoring a causative role for gut microbiota in the development of diabetes [86].

Further comparative studies of NOD mouse colonies with differing diabetes incidence have revealed that colonies with higher incidence have reduced microbial diversity and an elevated Firmicutes–Bacteroidetes ratio, as well as depleted populations of beneficial taxa such as *Coriobacteriaceae* and *Bifidobacteriaceae* [88]. *Akkermansia* species were absent in these diabetes-prone mice; however, supplementation with *Akkermansia muciniphila* in early life delayed diabetes onset and altered gut microbial communities, specifically reducing *Ruminococcus* populations. The latter genus has been associated with T1D in humans [88,89].

In rat models, preliminary studies comparing diabetes-prone (BBDP) and diabetes-resistant (BBDR) bio-breeding rats have provided the initial evidence for microbial associations with diabetes onset [90]. BBDP rats exhibit dysbiosis, which is characterized by an increase in pro-inflammatory genera, such as *Eubacterium*, *Ruminococcus*, and *Bacteroides*, alongside a reduction in the populations of potentially protective *Lactobacillus* and *Bifidobacterium* species [89,91,92].

A subsequent study by Valladares et al. identified a species of the *Lactobacillus* genus isolated from BBDR rats that was predominant in the prevention of T1D pathology [93]. The experimental interventions demonstrated strain-specific effects, with the administration of *L. johnsonii* significantly reducing the incidence of T1D in comparison to both control groups and animals that had been supplemented with *L. reuteri* [93].

In children with T1D, the TEDDY study constitutes the most extensive longitudinal investigation to date into the potential role of environmental factors in T1D, incorporating the microbiome [17]. That study identified probiotic strains, including *Lactobacillus rhamnosus*, *Lactococcus lactis*, and *Bifidobacterium dentium*, as potentially protective against the onset of T1D. Multiple independent studies across a range of regions have repeatedly observed disparate microbial profiles in T1D patients in comparison to healthy controls [92,94,95,96,97,98,99,100,101]. Numerous studies have reported a decrease in the Firmicutes–Bacteroidetes ratio in patients diagnosed with type 1 diabetes (T1D), though some studies employing duodenal biopsies have observed the converse [92,94,95]. This phenomenon may be attributable to the observation that duodenal biopsies exhibit elevated levels of *Firmicutes*, attributable to the predominance of *Streptococcus* in the proximal small intestine, whereas the microbiome in fecal samples is more representative of the colonic microbiota, with *Bacteroides* dominance. Furthermore, these studies demonstrate a decline in butyrate-producing bacteria, including *Clostridium*, *Roseburia faecis*, and *Faecalibacterium prausnitzii*, concomitant with an augmented abundance of *Bacteroides* and *Ruminococcus* in T1D patients [92,96,97,98]. With regard to members of the Bifidobacterium genus, the findings of two studies indicated reduced levels of T1D [91,92], while the results of a third study were contradictory [97].

Furthermore, the ongoing INNODIA study, a multicenter investigation of newly diagnosed T1D patients and unaffected family members, found that individuals with the most rapid declines in C-peptide levels—a marker of pancreatic β-cell function—had significantly reduced microbial richness [102]. An inverse correlation was also identified between *Bifidobacterium obeum* abundance and fluctuations in fasting C-peptide levels. These findings are consistent with the DIABIMMUNE cohort, which also found reduced microbial richness in infants and young children with HLA-conferred susceptibility to T1D [103].

Collectively, these studies imply that gut microbiota richness may function as a potential biomarker for disease progression. While animal models reveal causative links between specific taxa (e.g., Akkermansia) and diabetes protection, human studies demonstrate consistent patterns of reduced butyrogenesis and increased mucosal permeability. This functional convergence suggests that microbial metabolite production, rather than taxonomic composition alone, may drive T1D pathogenesis through dual mechanisms: (1) SCFA-mediated preservation of gut barrier integrity and (2) LPS-triggered pancreatic inflammation.

## 4. Potential Mechanisms Linking Gut Microbiota to T1D Pathogenesis

**Figure 2:**
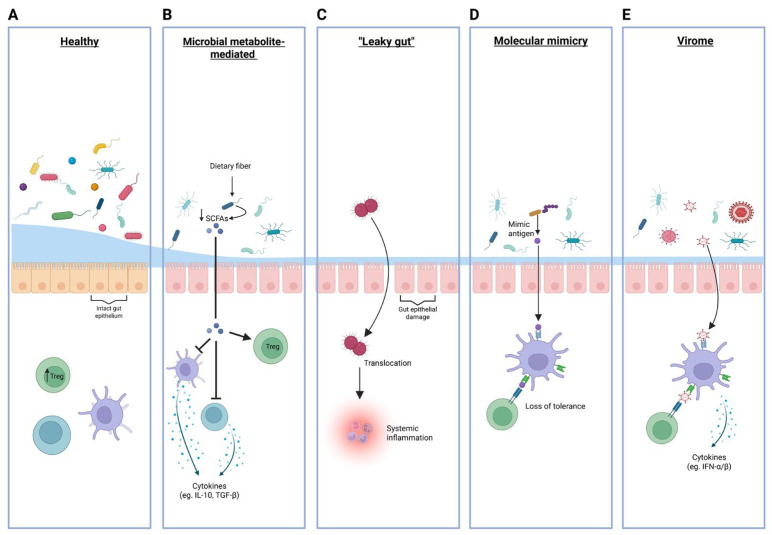
Schematic overview of mechanisms by which gut microbiota and virome influence immune regulation and the risk of autoimmunity in type 1 diabetes. Panels, from left to right, illustrate: (**A**) The healthy state, featuring a diverse commensal microbiota and intact gut epithelial barrier supporting regulatory T cell (Treg) induction and immune tolerance. (**B**) The role of microbial metabolites, where reduced dietary fiber fermentation leads to decreased production of short-chain fatty acids (SCFA; indicated by the downward arrow), resulting in impaired anti-inflammatory cytokine (IL-10, TGF-β) and Treg responses (**C**) ‘Leaky gut’, showing epithelial damage that permits microbial translocation across the intestinal barrier, leading to systemic inflammation. (**D**) Molecular mimicry, demonstrating activation of autoreactive lymphocytes via bacterial antigens resembling host proteins, resulting in loss of immune tolerance. (**E**) The virome, where viral sensing triggers type I interferon responses (IFN-α/β), potentially disrupting immune homeostasis and facilitating autoimmunity. Treg = regulatory T cell; SCFA = short-chain fatty acid; IFN-α/β = type I interferon.

The imbalance in gut microbial composition observed in T1D and the consequential perturbations in microbial metabolic capacity have been hypothesized to disturb the delicate balance between pro-inflammatory and anti-inflammatory pathways, thereby contributing to the autoimmune destruction of pancreatic β-cells. The influence of the gut microbiota on the pathogenesis of T1D is largely attributable to its effects on immune regulation, metabolic signaling, and gut barrier integrity (Figure 2).

### 4.1. Microbial Metabolite-Mediated Immune Regulation

Gut microbes are known to produce a myriad of microbial metabolites, the most notorious being short-chain fatty acids (SCFAs) [83]. Dietary fibers, which are not metabolized by host enzymes, are fermented by commensal bacteria in the large intestine to produce short-chain fatty acids, including succinate, acetate, propionate, and butyrate. SCFAs function as energy sources for enterocytes and exert anti-inflammatory effects on myeloid and lymphoid cells by activating G protein-coupled receptors or inhibiting histone deacetylases, leading to epigenetic modifications [104]. Butyrate has been shown to inhibit the pro-inflammatory cytokine production of macrophages and dendritic cells. In addition, it has been demonstrated to promote the differentiation and function of regulatory T cells (Tregs) by enhancing histone acetylation in the *FOXP3* gene [105,106,107,108,109]. However, in humans with longstanding T1D, oral butyrate supplementation has not shown significant effects on either innate or adaptive immunity, including no improvement in islet autoimmunity or β-cell function [110].

Another significant source of immunomodulatory molecules is the microbial metabolism of the essential amino acid dietary tryptophan, which results in the production of indole and its derivatives. These metabolites have been shown to activate the aryl hydrocarbon receptor (AhR), a transcription factor that plays a crucial role in regulating intestinal immunity and homeostasis [111]. Importantly for intestinal health, the activation of AhR in innate lymphoid cells and dendritic cells drives the secretion of IL-22, a cytokine that is critical for maintaining mucosal and epithelial barrier integrity. Furthermore, AhR activation promotes the differentiation of intraepithelial lymphocytes, which in turn produce anti-inflammatory cytokines such as IL-10 and TGF-β [111,112,113,114].

Recent studies have also highlighted the role of microbiome-derived bile acids in shaping immune responses relevant to T1D. Primary bile acids synthesized in the liver are converted by gut bacteria into secondary bile acids, which can modulate host metabolism and immune function through receptors such as TGR5 and FXR [115]. A longitudinal study by Lamichhane et al. demonstrated that children at risk for T1D who progressed to multiple islet autoantibodies exhibited distinct and persistently altered systemic bile acid profiles and gut microbial communities compared to controls, indicating that dysregulation of secondary bile acid metabolism and altered abundances of bile-acid-metabolizing microbes precede the development of islet autoimmunity [116]. These findings suggest that the gut microbiome–bile acid axis may contribute to the risk and pathogenesis of T1D by influencing immune homeostasis and metabolic signaling.

Overall, the capacity of commensal microbes to metabolize dietary components into immunomodulatory metabolites may shape the host immune landscape, both at the intestinal and systemic levels, and may be employed in targeted dietary interventions to promote immunotolerance in T1D.

### 4.2. “Leaky Gut”: Microbial Product Translocation to Systemic Circulation

In addition to metabolites, outer membrane vesicles (OMVs) from gut commensal bacteria have been shown to enter the bloodstream and prime type I interferon responses by delivering bacterial DNA or endotoxins, potentially enhancing antiviral immunity [117]. OMVs from *B. fragilis* have been implicated in exacerbating insulitis through activation of pattern recognition receptors, such as TLR9, and promoting inflammatory cytokine release, which can contribute to β-cell autoimmunity [118]. Nonetheless, not all OMVs exert pathogenic effects. OMVs derived from *Akkermansia muciniphila* have been reported to upregulate mucin-2 expression, thereby strengthening the intestinal barrier and potentially exerting protective effects against T1D by limiting systemic exposure to pro-inflammatory microbial products. Given their ability to cross the gut barrier and modulate immune responses at distant sites, OMVs and other microbial extracellular vesicles represent promising therapeutic targets in T1D.

### 4.3. Molecular Mimicry and Cross-Reactive Immune Responses

Another proposed mechanism is molecular mimicry. Indeed, it has been demonstrated that certain gut microbes can express antigens that bear a structural similarity to those of islet-derived epitopes that trigger cross-reactive immune responses. For instance, a sequence in *Parabacteroides distasonis* has been shown to mimic the insulin epitope insB:9-23, which is a key autoantigen in T1D. In the same vein, the colonization of NOD mice with *P. distasonis* accelerates T1D onset and aggravates insulitis. This is accompanied by an increased proportion of pathogenic CD8+ T cells, naive T cells, macrophages, and dendritic cells and a decrease in Treg cells in the spleen and pancreatic lymph nodes [119]. In another study, a mimic peptide from the Mgt protein of intestinal *Leptotrichia goodfellowii* was discovered to induce the activation of IGRP_206-214_-specific NY8.3 CD8 T cells in vitro and accelerate the development of T1D in NY8.3 NOD mice [120]. Conversely, microbial mimic peptides may also exert protective functions. For instance, the integrase protein of commensal microbes contains a low-avidity mimotope of IGRP_206-214_. As proof, colonization of germ-free NOD mice with integrase-competent or -deficient *Bacteroides* showed that integrase-derived peptide mimicry promotes the recruitment of diabetogenic CD8 T cells to the gut and suppresses colitis by killing dendritic cells loaded with microbial antigens [121].

### 4.4. Virome Contributions to T1D

Research over the past decade has increasingly implicated the human virome—the collection of all viruses in and on the body—as a significant factor in the development of type 1 diabetes (T1D), particularly in genetically susceptible individuals. Both eukaryotic viruses (infecting human cells) and bacteriophages (infecting bacteria) are involved, with evidence suggesting that alterations in the gut virome may precede and potentially influence the onset of autoimmune processes leading to T1D [122].

Numerous studies have highlighted the association between enteroviruses, particularly Coxsackie B viruses (CVB), and the onset of T1D. High viral titers and multiplicity of infection, rather than specific viral phenotypes, appear to determine the diabetogenic potential of enteroviruses in genetically predisposed hosts. Animal models demonstrate that infection with various CVB strains can trigger rapid diabetes onset in mice with pre-existing insulitis. Compelling human evidence includes the detection of enterovirus infection in pancreatic tissue from adults with recent-onset T1D and enhanced islet antiviral immune responses, as shown by the nPOD Virus Group.

Longitudinal studies of children at risk for T1D have revealed that those who develop islet autoimmunity exhibit a less diverse intestinal virome compared to controls. Specifically, a lower abundance of *Circoviridae*-related sequences and reduced diversity of bacteriophages were observed in cases versus controls. These alterations in the gut virome precede the development of autoimmunity, suggesting a potential contributory or predictive role [123,124]. Notably, while enteroviruses, kobuvirus, parechovirus, parvovirus, and rotavirus are frequently detected in the gut, only enteroviruses have shown a consistent association with T1D onset [123].

A recent randomized controlled trial on children and adolescents with new-onset T1D showed that antiviral treatment (pleconaril and ribavirin) can preserve residual insulin production for at least one year after diagnosis, compared to placebo [63]. This suggests that targeting persistent viral infections, especially enteroviruses, may slow disease progression and preserve β-cell function in early T1D. However, longer-term follow-up studies (up to three years) indicate that the protective effect on insulin production may diminish over time, with no statistically significant difference between treated and placebo groups at two and three years [125]. These findings highlight the need for further research to optimize timing, duration, and patient selection for antiviral interventions.

## 5. Gut Microbiome Therapies: Fecal Microbiota Transplantations

Fecal microbiota transplantation (FMT) has emerged as an important experimental tool in preclinical research and is also being explored as a therapeutic strategy to mitigate or prevent T1D onset [29]. FMT involves the transfer of donor fecal matter (in aqueous solution form) into a recipient’s intestinal tract in order to alter the gut microbial composition of the recipient, with the aim of conferring health benefits [126]. FMT is primarily designed to regulate the gut microbial balance, reduce the dominance of unfavorable or pathogenic microbial species, and promote colonization by favorable or symbiotic species, including production of metabolites such as SCFAs [127]. Fecal transplants contain not only viable, dead, or damaged bacteria but also fungi, archaea, protists, and viruses. Other components include colonocytes, mucus, proteins, fat, and metabolites such as bile acids and SCFAs [128,129]. Prior to FMT, donor feces are mixed with water or saline, and insoluble substances are discarded by filtration. The type of donor feces and route of administration of FMT can vary. Donor feces can be either allogeneic or autologous. Allogeneic FMT uses stool from a healthy, rigorously screened donor, and this can come from one or multiple donors. In contrast, autologous FMT involves the patient’s own stool, ideally collected during a healthy state and later introduced to restore baseline microbiota [130]. Using the patient’s own stool offers advantages, as it avoids the need for challenging donor recruitment and rigorous screening, and it minimizes the risk of transmitting infections, pathogens, or other disease phenotypes [131,132]. Additionally, the efficacy of autologous FMT may be increased due to better engraftment, as demonstrated in the treatment of recurrent *Clostridioides difficile* infections in hematological diseases [133]. FMT can be administered through several routes, which are generally classified as upper or lower gastrointestinal approaches. Common upper gastrointestinal routes include nasogastric, nasoduodenal, and naso-jejunal tubes, as well as oral capsules. Lower gastrointestinal routes involve colonoscopy, sigmoidoscopy, or rectal enema [134]. No single route has been universally established as superior, and the optimal method may vary depending on the patient and condition being treated, considering the level of invasiveness, comfort, compliance, and cost-effectiveness.

### 5.1. FMT in T1D Research: Current Findings and Potential

In preclinical T1D research, FMT has served as a valuable experimental tool for demonstrating the gut microbiota’s role in disease pathogenesis, proving both transmissibility of the disease phenotype through the gut microbiome and the therapeutic potential of FMT (Table 2) [79,86].

In a case report, a 24-year-old T1D patient with malnutrition due to gastrointestinal symptoms received allogeneic FMT from a healthy donor via the naso-jejunal tube. Along with a shift in gut microbiome composition toward that of the healthy donor, glycemic parameters improved. However, this improvement may also be attributable to the relief of gastrointestinal symptoms [135]. One larger RCT evaluated the effects of FMT on adults with newly diagnosed T1D (within six weeks of onset), using either an allogeneic (healthy donor, n = 11) or an autologous approach (n = 10). Participants received three consecutive FMTs via a nasoduodenal tube over four months, with a follow-up of 12 months. Remarkably, the autologous FMT group exhibited stable fasted and stimulated C-peptide levels, indicating significant preservation of endogenous beta cell function, but this was not observed following allogeneic FMT. Subsequent autologous FMTs were associated with alterations in gut microbial composition and plasma metabolite profiles, including increased levels of fecal *Desulfovibrio piger*, plasma phospholipids, and tryptophan derivatives. Additionally, FMT was found to influence the distribution of the activated T cell subsets CD4+ CXCR3+ and CD8+ CXCXR3+, which was inversely correlated to both C-peptide levels and the abundance of *D. piger* [81]. Although no true placebo control (lavage without fecal transfer) was included, these findings suggest that autologous FMT may contribute to maintaining beta cell function in the early stages of T1D by influencing the gut microbiota composition, host metabolism, and immune cell dynamics, with no significant safety concerns observed.

Encapsulated FMT is gaining prominence as a preferred method for microbiota transfer, largely due to its non-invasive nature, improved patient comfort, and higher acceptability compared to traditional invasive procedures, such as nasoduodenal tubes or colonoscopies. Fecal microbiota capsules (FMCs) are particularly advantageous for use with vulnerable populations, including young children at risk of, or newly diagnosed with, T1D, as they can be self-administered and facilitate more regular and prolonged treatment, even in home settings [139]. Freeze-dried FMCs have already been shown to be safe, easy to use, and well tolerated in the treatment of *C. difficile* infection [140]. In a small study, autologous FMT was performed on two adolescent patients with T1D and a disease duration of one year. The FMT protocol differed for the two patients, as one patient received trans-nasal FMT at week 1, followed by oral FMT capsules at weeks 6 and 8, with a follow-up of 30 weeks, and the other patient received one FMT with oral capsules at week 1, with a follow-up of 10 weeks. Glycemic parameters improved together with increased diversity of fecal microbiota comparing post- and pre-transplantation, although the results should be cautiously interpreted with the small sample size [136]. Encapsulated FMT in T1D was also studied in the context of diabetic gastroenteropathy, studying safety, feasibility, and efficacy at 4 weeks after treatment in a randomized, double-blinded, placebo-controlled pilot trial. T1D patients with gastroenteropathy were randomized to receive either encapsulated allogeneic FMT or a placebo. Encapsulated FMT was proven to be safe, as no serious adverse events attributable to FMT were observed. Patients who received FMT demonstrated a significantly greater reduction in gastrointestinal symptoms, as measured by validated irritable bowel syndrome questionnaires, and reported an improved quality of life compared to those who received a placebo. Additionally, FMT recipients exhibited increased gut microbial alpha and beta diversity, suggesting enhanced microbial richness and community composition following treatment [137]. Our research group has recently published a paper on the effect of encapsulated autologous FMTs on the preservation of residual beta cell function in patients who have been recently diagnosed with T1D (within 0.5–3.5 years; n = 10) [138]. This pilot study provides promising preliminary evidence that encapsulated autologous fecal microbiota capsules could be a feasible and safe adjunct therapy to preserve endogenous insulin production in type 1 diabetes. However, further research with larger sample sizes is needed to validate efficacy.

### 5.2. Future of FMT in T1D: Challenges and Considerations

Overall, FMT holds great promise as an adjunctive therapy for T1D, as these small clinical studies demonstrate that FMT can improve glycemic control, modulate immune responses, and potentially help preserve residual pancreatic beta cell function. Despite these encouraging findings, several questions and technical challenges remain before FMT can be widely adopted in clinical practice. Although FMT is generally considered safe, with adverse events being rare, there are still risks involved, such as the transmission of multi-resistant bacteria [141]. To mitigate these risks, a rigorous donor screening program is essential, supported by the guidelines of the European Society for Microbiology and Infectious Diseases and the European FMT Working Group [142]. This will mean that only a small proportion of potential donors will ultimately be deemed eligible, with frequent repetition required to maintain safety standards. However, the study by De Groot et al. found that favoring autologous FMT could help to overcome this challenge [81]. Another key consideration is the precision of the procedure. Current approaches to donor selection and transplantation are often random, with high heterogeneity in donor feces. This can potentially limit efficacy and introduce irrelevant, or even harmful, bacteria that could negatively impact gut permeability and immune function [143]. A more personalized approach to FMT, such as matching donor and recipient microbiomes to ensure better compatibility [144] or using a defined bacterial mixture, could improve the effectiveness and reproducibility of treatment outcomes. Synthetic stool, for example, has a precisely known composition, is free from viruses and other pathogens, and was demonstrated to be a safe and reproducible alternative in a small proof-of-principle study for antibiotic-resistant *C. difficile* infection [145]. Moreover, the ideal treatment regimen—including the optimal site of FMT administration, duration of FMT, number of sessions, and intervals between treatments—also remains to be established, and the longevity of FMT’s effects is still unclear. Long-term studies are also needed to fully assess the safety and effectiveness of FMT in T1D management. By contrast, FMT is now a thoroughly validated and widely utilized treatment for recurrent *Clostridioides difficile* infection, with extensive data supporting its long-term safety and efficacy [146]. In T1D, however, FMT remains experimental, and definitive long-term studies are still needed to determine its safety, optimal protocols, and durability of effects. Addressing these challenges is essential for transitioning FMT from an experimental therapy to a standard treatment for T1D.

## 6. Microbial Metabolite-Driven Therapies in T1D

Shifting the therapeutic focus from the bacterial species responsible for producing beneficial metabolites to the metabolites themselves is a promising approach to managing and potentially modifying T1D. This metabolite-centric approach aims to modulate the functional outputs of the gut microbiome directly, bypassing the variability and complexity associated with manipulating live microbial organisms [147]. As previously mentioned, metabolites such as SCFAs can have beneficial metabolic and immunomodulatory effects [148]. Postbiotics, which are defined as “preparations of inanimate microorganisms and/or their components that confer a health benefit on the host”, can be used alongside dietary interventions as metabolite-driven therapies (Table 3) [147].

SCFA supplementation has been tested in two RCTs. In a placebo-controlled, double-blind crossover study, 30 individuals with long-standing T1D received oral sodium butyrate (4 g/day) for one month. This intervention resulted in only minor alterations to the composition of the gut microbiota and no significant changes in beta cell function or glycemic control, as assessed by stimulated C-peptide levels and HbA1c, respectively. No changes in the innate immune phenotype were observed. Innate immune system phenotyping and functional assays showed no significant changes compared to the placebo group. However, butyrate supplementation was associated with a significant reduction in IA2-specific CD8+ T cells, while CD8+ T cells targeting other beta cell epitopes remained unaffected [110]. A subsequent larger trial (n = 53), which administered 3.6 g of sodium butyrate or a placebo daily for 12 weeks, likewise found no improvements in inflammatory markers, renal parameters, HbA1c, albuminuria, or intestinal inflammation [149].

Recent research has explored alternative strategies for delivering SCFAs by modifying resistant starches with acetate and butyrate. Building on earlier evidence that high-amylose maize starch modified with acetate and butyrate (HAMSAB) improves glycemia and reduces pancreatic insulitis in animal models [154], two clinical trials were conducted on patients with long-standing and recent-onset T1D [155,156]. In the first trial, adults with long-standing T1D received HAMSAB for six weeks, with follow-up extending to twelve weeks. The intervention led to notable increases in fecal and plasma SCFA levels, which persisted even after a 6-week washout period. These changes were accompanied by shifts in gut microbiota composition, including increased abundance of *Bacteroides uniformis* and *Parabacteroides* species, alongside a reduction in *Eubacterium ramulus*, *Eubacterium eligens*, and *Coprococcus comes.* Immunologically, the HAMSAB diet appeared to create a more tolerogenic environment, as pro-inflammatory cytokines decreased and regulatory markers on T and B cells increased, such as TIGIT and CTLA-4 on T cells and CD86 and MHC-I on marginal zone-like B cells. Notably, these immunological benefits were sustained after the intervention ended. Despite the positive effects on the microbiome and immune system, the HAMSAB supplementation did not significantly improve glycemic control or beta cell function, as measured by fasting glucose and C-peptide levels. However, those individuals with the highest SCFA concentrations did show better glycemic outcomes, suggesting a possible dose–response relationship [155]. A pilot crossover study exploring HAMSAB supplementation for four weeks in adolescents with recent-onset T1D (<2 years) also found no significant effects on glycemia or beta cell function compared to a normal diet, though it did report reduced activation of mucosal-associated invariant T (MAIT) cells, which play a role in mucosal immunity [156]. The limited clinical benefits of both oral supplementation of butyrate and HAMSAB supplementation may be attributable to the timing of intervention, given the inclusion of long-standing T1D, and the relatively short duration of the treatment (4 weeks). In this context, a larger phase Ib double-blind, placebo-controlled trial (NCT06057454) is currently running to evaluate the effects of the HAMSAB diet on patients with newly diagnosed T1D.

Diet-derived gut microbiota-produced metabolite-driven therapeutic approaches can also be achieved through dietary strategies that increase the availability of substrates for gut microbial fermentation and the production of SCFAs. These strategies involve the consumption of dietary fibers such as inulin, fructo-oligosaccharides (FOS), and galacto-oligosaccharides (GOS), which are also known as prebiotics [157]. The intake of dietary fibers may contribute to glycemic control, as a higher intake of dietary fiber is independently associated with a higher time spent in the euglycemic range in T1D patients (n = 470) [150]. Furthermore, an 18-month RCT behavioral nutrition intervention study involving T1D patients (n = 136) showed that high fiber intake was associated with better glycemic control, as assessed by the 1,5-anhydroglucitol assay, as well as CGM metrics [151]. Additionally, a 24-week high-fiber diet composed of natural foods, compared to a low-fiber diet, significantly reduced the mean daily blood glucose and the number of hypoglycemic episodes in T1D patients (n = 63) [152]. Interestingly, an RCT testing the effects of 12 weeks of oligofructose-enriched inulin supplementation found significant preservation of pancreatic beta cell function compared to placebo in children with T1D (n = 38). Here, C-peptide levels were significantly higher after prebiotic supplementation, alongside gut microbial changes and significantly improved gut permeability, as measured by the lactulose/mannitol test. However, no significant changes in glycemic marker HbA1c or inflammatory markers were observed after 12 weeks [153]. It should be noted, however, that in the Finnish Type 1 Diabetes Prediction and Prevention (DIPP) study, a prospective birth cohort showed that the development of islet autoimmunity in children with a genetic predisposition to T1D was associated with the intake of non-specified dietary fibers (hazard ratio, 1.41; 95% CI, 506 1.10–1.81) [158]. This suggests that not all fibers may be beneficial in autoimmune diabetes and that the timing of fiber intake (childhood vs. adulthood) may drive different effects, although this provides no information about overall production of SCFAs or the influence of produced metabolites on islet autoimmunity.

## 7. Discussion

Recent advances in research on type 1 diabetes (T1D) have revealed a complex interplay between immune dysregulation and environmental factors, particularly the gut microbiome, in the pathogenesis of the disease and the identification of therapeutic opportunities. The advent of novel therapeutic interventions has led to the development of multifaceted approaches that harness the potential of immune cell engineering and targeted immunomodulation. Prominent examples include CAR-Treg therapy and JAK-STAT inhibitors. In addition to these immune-based interventions, novel therapeutic strategies have emerged that focus on restoring microbiome diversity and function. This includes the administration of fecal microbiota transplantation (FMT) and metabolite supplementation.

Immune-based interventions, including adoptive regulatory T cell transfer and agents targeting T cell co-stimulation or cytokine signaling, have demonstrated the potential to delay the onset of T1D and partially preserve beta cell function. Nevertheless, limitations, such as variable efficacy, safety concerns (e.g., risk of infection or malignancy and off-target effects of genetic engineering), and accessibility continue to restrict widespread clinical adoption. Moreover, the inherent heterogeneity of this disease and the necessity for personalized therapy models pose persistent challenges.

The gut microbiome has emerged as a promising therapeutic target due to its profound effects on both local and systemic immune homeostasis. A body of research has emerged from both animal and human studies that suggests a link between type 1 diabetes (T1D) and reduced microbial diversity, along with consistent functional shifts, particularly in short-chain fatty acid (SCFA) production and epithelial barrier integrity. FMT, particularly autologous transplantation, in addition to dietary and synthetic approaches aimed at enhancing beneficial microbial metabolites, has demonstrated the potential to modulate immune responses and glycemic indices in preclinical and early clinical trials. While these interventions have been shown to modify the composition of the microbiome, metabolic profiles, and specific immune markers, the extent of sustained clinical benefits on glycemic control and preservation of beta cells remains limited.

It is important to note that further research is required to establish the durability of these effects, to define optimal delivery protocols, to ascertain the safety of donor material, and to determine the risk of adverse events (such as pathogen transmission). These issues must be addressed through larger and longer-term trials.

Furthermore, metabolite-centric interventions, such as butyrate or resistant starch supplementation, have thus far resulted in immunological effects but minimal or inconsistent improvements in glucose homeostasis or C-peptide levels. These limitations may pertain to the timing of intervention, the heterogeneity of the underlying microbiome, or the necessity for concomitant modulation of other disease-relevant pathways. Intriguingly, high-fiber dietary interventions and certain prebiotics have been associated with improved glycemic outcomes and may be especially useful in combination with immune-based therapies.

While initial combinations of dietary interventions and immune therapies have shown promise, the future lies in integrative personalized approaches that combine next-generation immunotherapies with microbiome-targeted strategies tailored to the patient’s characteristics in immunoprofiles and intestinal microbial signatures.

Future research should focus on elucidating mechanistic links between host genetics, environmental triggers, and microbiome-immune cross-talk in T1D. The development of strategies to optimize FMT (including donor–recipient matching, defined bacterial consortia, and synthetic stool alternatives) is of critical importance, as is the advancement of the precision of metabolite supplementation and the design of rational combination therapies that harness both immune and microbial targets. In order to assess the safety, durability, and real-world effectiveness of these next-generation approaches, it is essential to conduct long-term, multicenter clinical trials. The integration of multi-omics profiling, biomarker-guided patient stratification, and systems immunology will further enable the transition from empirical, one-size-fits-all regimens to durable, personalized therapies for individuals with or at risk of T1D.

## 8. Materials and Methods

This narrative review synthesizes data from preclinical and clinical studies on innovative targeted therapies for type 1 diabetes (T1D), focusing on microbiome-based immunomodulation and metabolite-driven interventions. The literature was identified exclusively through systematic searches of the PubMed database up to September 2025. Search terms included “type 1 diabetes,” “microbiome,” “immune modulation,” “immunotherapy”, “FMT”, “microbiota composition”, and “microbial metabolites (e.g., short-chain fatty acids)”, among others.

Only peer-reviewed articles published in English were considered. Both original research and review articles were included to provide a comprehensive overview of current and emerging therapeutic strategies. Details of ongoing and completed clinical trials were cross-referenced with available clinical trial registries when applicable.

Generative artificial intelligence (GenAI) tools were used solely for language editing and formatting assistance, without contributing to the scientific content or analysis of this manuscript.

## 9. Conclusions

More mechanistic and clinical research studies are warranted to unravel the mechanisms and modifiable pathways that underpin T1D development, as well as to identify the environmental factors that trigger T1D onset or modulate its progression. In this regard, the gut microbiota provides new immune-modulatory mechanisms and therapeutic leads in T1D. Future research should focus on improving FMT therapies, enhancing bacteria engraftment, and investigating the potential therapeutic benefits of beneficial microbial metabolites beyond SCFA. The combination of microbiome-targeting strategies and immunotherapy may provide long-term preservation of beta cell function by re-establishing intestinal and systemic immune homeostasis.

## Figures and Tables

**Table 1:** Detailed summary of key immune-targeted clinical and preclinical studies in type 1 diabetes pathophysiology and therapy.

Intervention	Population Description	Main Outcomes	References
Teplizumab (anti-CD3 mAb)	At-risk individuals with familial/genetic risk; newly diagnosed T1D patients (within weeks to months of onset)	Delayed T1D onset by ~2 years; preserved beta cell function; no HbA1c improvement	[14,31,32,36]
Abatacept (CD80/CD86 blockade)	New-onset T1D patients, typically <3 months post-diagnosis	Slowed beta cell decline over 2 years; reduced memory and follicular helper T cells; no delay in progression to glucose intolerance in at-risk individuals	[33,34,35,37]
Regulatory T cell (Treg) therapies	New-onset T1D children and NOD mouse models	Defective function in Treg from T1D patients; adoptive transfer reversed diabetes in mice; low-dose IL-2 expanded Tregs with mixed effects	[38,39,40,41,42,43,44,45,46,47,48]
CAR-Treg therapy	Preclinical murine models (NOD mice)	Antigen-specific CAR-Tregs prevented diabetes onset; induced broad immunosuppression against islet-specific T cells	[49,50,51]
JAK-STAT pathway inhibitors	New-onset T1D patients (first 12 weeks); rare monogenic autoimmune T1D cases	Baricitinib preserved beta cell function and reduced inflammation; ruxolitinib reversed autoimmune diabetes in a mutation case	[52,53,54,55]
Beta cell neoantigens	Human pancreatic tissues; T1D patient samples	Post-translational modifications create neoantigens that trigger autoreactive T cells	[56,57,58,59,60,61]
Environmental triggers	Birth cohorts; genetically at-risk children; general population	Viral infections, diet (gluten, vitamin D), and hygiene hypothesis influence T1D risk	[17,36,37,62,63,64,65,66]

**Table 2:** Summary of main clinical studies using fecal microbiota transplantation as an intervention for the treatment of type 1 diabetes.

Intervention	Population	Main Changes	Reference
Allogeneic FMT	Twenty-year-old T1D patient with malnutrition and GI symptoms (n = 1)	Shift in gut microbiome toward donor; improved glycemia (possibly due to symptom relief)	[135]
Allogeneic and Autologous FMT	Adults, newly diagnosed T1D (within 6 weeks) (n = 21)	Autologous group maintained stable C-peptide, with changes in microbiome, metabolites, and immune cells; allogeneic group did not	[81]
Autologous Encapsulated FMT	Adolescents with 1-year T1D (n = 2)	Improved glycemia and increased gut microbial diversity	[136]
Encapsulated Allogeneic FMT	T1D patients with gastroenteropathy	Reduction in GI symptoms; increased gut microbial diversity; improved quality of life	[137]
Autologous Encapsulated FMT (Ongoing)	Recently diagnosed T1D (0.5–3.5 years)	No significant decrease in beta cell function, suggesting the treatment may stabilize beta cell function	[138]

**Table 3:** Summary of main clinical studies investigating microbial metabolite-driven therapies in type 1 diabetes.

Intervention	Population	Main Changes	Reference
Oral sodium butyrate 4 g/day for 1 month	Individuals with long-standing T1D (n = 30)	Minor microbiota changes; no improvement in beta cell function or glycemic control; reduced IA2+ CD8+ T cells	[110]
Oral sodium butyrate 3.6 g/day for 12 weeks	Individuals with long-standing T1D (n = 53)	No changes in inflammation or glycemic markers	[149]
HAMSAB, 6-week + 6-week washout	Adults with long-standing T1D	Increased fecal and plasma SCFA; microbial shifts; immune modulation; no glycemic improvement	[147]
HAMSAB, 4-week pilot crossover study	Adolescents with recent-onset T1D	Immune changes (reduced MAIT activation); no glycemic change	[148]
Diabetic diet rich in inulin, FOS, GOS (prebiotics)	T1D patients (n = 470)	Associated with better glycemic control	[150]
Increased dietary fiber intake (inulin, FOS, GOS)	T1D patients (n = 136)	Improved glycemic control by CGM and other metrics	[151]
High fiber natural foods diet, 24 weeks	T1D patients (n = 63)	Reduced blood glucose and hypoglycemic episodes	[152]
Oligofructose-enriched inulin 12 weeks	Children with T1D (n = 38)	Preserved beta cell function; microbial changes; improved gut permeability; no HbA1c changes	[153]

## Data Availability

No new data were created or analyzed in this study. Data sharing is not applicable to this article.

## Abbreviations

The following abbreviations are used in this manuscript:

AID	Automated Insulin Delivery
APC	Antigen-Presenting Cell
CAR-Treg	Chimeric Antigen Receptor Regulatory T cells
CGM	Continuous Glucose Monitoring
DIPP	Type 1 Diabetes Prediction and Prevention
FMT	Fecal Microbiota Transplantation
FOXP3	Forkhead Box P3 (Treg transcription factor)
GADA	Glutamic Acid Decarboxylase Antibodies
HbA1c	Hemoglobin A1c (glycemic control marker)
HLA	Human Leukocyte Antigen
IAA	Insulin Autoantibodies
IA-2A	Islet Antigen-2 Antibodies
JAK-STAT	Janus Kinase–Signal Transducer and Activator of Transcription Pathway
MAIT	Mucosal-Associated Invariant T cells
MODY	Maturity Onset Diabetes of the Young
MHC	Major Histocompatibility Complex
NOD	Non-Obese Diabetic (Mouse Model)
OMV	Outer Membrane Vesicles
RCT	Randomized Controlled Trial
SCFA	Short-Chain Fatty Acids
STAT	Signal Transducer and Activator of Transcription
TCR	T Cell Receptor
Tfh	Follicular Helper T cells
Th1, Th17	T Helper Cell Subsets
TIGIT	T Cell Immunoreceptor with Ig and ITIM Domains
ZnT8	Zinc Transporter 8
